# Serum Osteopontin Is Associated with Cardio-Ankle Vascular Index-Defined Arterial Stiffness in Patients with Type 2 Diabetes Mellitus: A Cross-Sectional Study

**DOI:** 10.3390/diagnostics16203300

**Published:** 2026-10-11

**Authors:** Cing-Yu Liang, Jer-Chuan Li, Du-An Wu, Bang-Gee Hsu

**Affiliations:** 1 Department of Internal Medicine, Hualien Tzu Chi Hospital, Buddhist Tzu Chi Medical Foundation, Hualien 97004, Taiwan; 2 Division of Metabolism and Endocrinology, Buddhist Tzu Chi General Hospital, Hualien 97004, Taiwan; 3 School of Medicine, Tzu Chi University, Hualien 97004, Taiwan; 4 Division of Nephrology, Hualien Tzu Chi Hospital, Buddhist Tzu Chi Medical Foundation, Hualien 97004, Taiwan

**Keywords:** type 2 diabetes mellitus, osteopontin, cardio-ankle vascular index, peripheral arterial stiffness, logistic regression, bootstrap validation

## Abstract

**Background:** Peripheral arterial stiffness (PAS) is common in patients with type 2 diabetes mellitus (T2DM). Despite osteopontin (OPN) being linked to structural alterations in the vasculature, its specific association with the cardio-ankle vascular index (CAVI) as an indicator of PAS is still unclear. **Methods:** A total of 120 patients diagnosed with T2DM were enrolled in this cross-sectional study. Participants were stratified into the PAS group (CAVI ≥ 9.0) and the control group (CAVI < 9.0) based on the diagnostic criterion of CAVI ≥ 9.0. Circulating OPN concentrations were determined via a commercial enzyme-linked immunosorbent assay kit. **Results:** PAS was present in 72 (60.0%) participants. Relative to the control group, individuals with PAS were older (*p* < 0.001) and had higher fasting glucose (*p* = 0.015), glycated hemoglobin (*p* = 0.006), urine albumin-to-creatinine ratio (*p* = 0.007), and OPN (*p* < 0.001) levels, whereas their high-density lipoprotein cholesterol (HDL-C) concentrations were markedly lower (*p* = 0.021). Spearman’s rank correlation demonstrated a significant positive association between serum OPN concentrations and bilateral CAVI measurements (both *p* < 0.001). In multivariable Firth logistic regression, elevated serum OPN concentrations were independently predictive of PAS (adjusted odds ratio [aOR] = 1.035; 95% confidence interval [CI]: 1.012–1.066; *p* = 0.001), along with older age and lower HDL-C levels. These associations were consistent across conventional and penalized logistic regression analyses. Receiver operating characteristic curve analysis revealed that OPN alone achieved an area under the curve (AUC) of 0.748, whereas the model combining OPN, age, and HDL-C achieved an AUC of 0.831. **Conclusions:** Higher serum OPN levels were independently associated with PAS in patients with T2DM, particularly when combined with age and HDL-C.

## 1. Introduction

Type 2 diabetes mellitus (T2DM) is a major global health challenge associated with a substantially increased risk of cardiovascular (CV) morbidity and mortality [1]. Cardiovascular disease (CVD) remains the principal contributor to mortality in individuals diagnosed with T2DM, who have an approximately two- to fourfold higher risk of CV events than individuals without diabetes [1]. Although traditional risk factors, including hyperglycemia, dyslipidemia, and hypertension, contribute substantially to this increased risk, they do not fully explain the accelerated vascular aging observed in this population [2]. Growing evidence indicates that arterial stiffness is a key pathophysiological mechanism and an independent predictor of adverse CV outcomes [3]. Evaluated via the cardio-ankle vascular index (CAVI), peripheral arterial stiffness (PAS) serves as an established surrogate of vascular wall integrity, capturing integrated structural and functional damage across the arterial tree from the ascending aorta down to the ankle arteries [4]. Derived from the stiffness parameter β of the arterial wall, CAVI is designed to minimize the influence of blood pressure at the time of measurement, thereby overcoming a major limitation of traditional pulse wave velocity (PWV) measurements [4]. Enhanced arterial stiffness exacerbates systolic hypertension and cardiac afterload, significantly increasing the incidence of myocardial infarction, heart failure, and cardiovascular death in the T2DM population [5].

Osteopontin (OPN), a multifunctional matricellular phosphoprotein and proinflammatory cytokine, has emerged as an important mediator of diabetic macrovascular complications [6,7]. Initially identified as a regulator of bone metabolism, OPN is now recognized for its diverse roles in vascular remodeling and calcification [6,7]. In T2DM, chronic hyperglycemia and oxidative stress upregulate OPN expression in vascular smooth muscle cells and macrophages, promoting a proinflammatory environment that accelerates arterial wall degradation [6,7]. Accumulating clinical evidence indicates that heightened circulating OPN concentrations correlate strongly with the extent and progression of coronary artery disease while also serving as a robust predictor of major adverse cardiovascular events [8]. Despite increasing evidence supporting the role of OPN in systemic atherosclerosis, its specific association with PAS in patients with T2DM remains poorly defined. Determining whether serum OPN levels are independently associated with PAS, as measured by CAVI, may identify a clinically informative marker reflecting concurrent subclinical vascular damage. Therefore, the present study was conducted to evaluate the relationship between circulating OPN concentrations and PAS among individuals with T2DM.

## 2. Materials and Methods

### 2.1. Patients

In this cross-sectional investigation, we enrolled 120 individuals with T2DM who received their diagnoses at a medical center located in eastern Taiwan from January to August 2021. Standardized protocols were followed for blood pressure (BP) assessment; participants rested in a seated position for a minimum of 30 min prior to morning recordings of systolic blood pressure (SBP) and diastolic blood pressure (DBP). To minimize confounding by overt macrovascular disease, patients with acute coronary syndrome, decompensated heart failure, lower-extremity amputation, cancer, active infections, liver cirrhosis, chronic obstructive pulmonary disease, or refusal to provide informed consent were excluded. The Institutional Review Board/Research Ethics Committee of Hualien Tzu Chi Hospital formally reviewed and approved the study protocol (IRB108-219-A). Signed informed consent was secured from each participant before enrollment. Detailed clinical profiles, baseline cardiovascular comorbidities, and comprehensive treatment exposures across antihypertensive, lipid-lowering, and antidiabetic pharmacotherapies were systematically extracted from medical records. However, quantitative lifestyle behaviors (such as structured physical activity and cumulative cigarette smoking exposure), medication adherence, and exact diabetes duration from initial diagnosis were not systematically captured at enrollment.

### 2.2. Anthropometric Analysis

Anthropometric evaluations were conducted under standardized conditions (barefoot and wearing light indoor attire), recording weight to within 0.5 kg and height to within 0.5 cm. Anthropometric parameters included waist circumference, recorded at the midpoint between the lower rib edge and the iliac crest in a standing posture with hands placed on the hips, and body mass index (BMI), which was derived by dividing weight (kg) by height squared (m^2^).

### 2.3. Biochemical Investigations

Following collection, fasting blood specimens were promptly centrifuged at 3000× *g* for 10 min; the isolated serum aliquots were subsequently stored at −80 °C pending biochemical analysis. An automated chemistry analyzer (Advia 1800; Siemens Healthcare GmbH, Erlangen, Germany) was utilized to assess serum concentrations of fasting glucose, HbA1c, blood urea nitrogen, creatinine, lipid profiles (total cholesterol [TCH], triglycerides [TG], high-density lipoprotein cholesterol [HDL-C], low-density lipoprotein cholesterol [LDL-C]), albumin, total calcium, and inorganic phosphorus. The urine albumin-to-creatinine ratio (UACR) was determined from random single-void urine specimens. Serum osteopontin and intact parathyroid hormone were assayed via commercial ELISA kits (OPN: BMS2066, eBioscience Inc., San Diego, CA, USA; iPTH: NM59041, IBL International, Hamburg, Germany) in accordance with the manufacturers’ instructions [9]. The intra-assay and inter-assay coefficients of variation were 6.7% and 6.1%, respectively, for OPN and 4.2% and 6.1%, respectively, for intact parathyroid hormone. The 2021 Chronic Kidney Disease Epidemiology Collaboration (CKD-EPI 2021) equation was utilized to determine the estimated glomerular filtration rate (eGFR).

### 2.4. BP and CAVI Measurements

After blood collection, participants rested in the supine position for 30 min. An experienced operator measured morning BP using an automatic oscillometric device applied to the upper arm. Systolic and diastolic blood pressures in the right brachial artery were evaluated three times with a 5 min resting gap between recordings, and their mean was calculated for analysis. In accordance with the Eighth Joint National Committee (JNC 8) guidelines, hypertension was defined as an SBP ≥ 140 mmHg, a DBP ≥ 90 mmHg, or current use of antihypertensive pharmacotherapy within the past 2 weeks. CAVI was measured using a waveform analyzer (VaSera VS-1000; Fukuda Denshi, Tokyo, Japan) according to the manufacturer’s recommendations. Participants remained in the supine position and rested for 10 min before simultaneous electrocardiogram and phonocardiogram recordings, BP measurements of the upper and lower extremities, and automatic measurements of PWV and CAVI were obtained [10]. By combining brachial blood pressure with heart-to-ankle PWV (the transit time of the pulse wave from the aortic valve to the ankle), CAVI quantitatively evaluates regional arterial elasticity [10]. Its mathematical formulation is based on the Bramwell–Hill equation describing the relationship between PWV and arterial volume change and is ultimately derived from the arterial stiffness parameter β described by Hayashi et al. [11]. The detailed calculation method for CAVI has been described previously by Miyoshi et al. [10]. Following the clinical recommendations of the Japan Society for Vascular Failure, a CAVI cutoff value of ≥9.0 was applied to identify the presence of PAS and vascular dysfunction in our cohort [12], which has also been widely adopted and clinically applied in East Asian and Taiwanese cohorts to define established subclinical arteriosclerosis. Participants with a CAVI ≥ 9.0 on either side were classified into the PAS group, whereas those with bilateral CAVI values < 9.0 were assigned to the control group.

### 2.5. Statistical Analysis

Data processing and statistical evaluations were executed via SPSS (version 25.0; IBM Corp., Armonk, NY, USA) in conjunction with R software (version 4.2.2; R Foundation for Statistical Computing, Vienna, Austria). The Kolmogorov–Smirnov test was employed to evaluate the normality of continuous data distribution. Continuous parameters adhering to a normal distribution were reported as mean ± standard deviation (SD); intergroup comparisons were performed via two-sample Student’s *t*-tests. For continuous parameters exhibiting a skewed distribution, data were reported as the median (interquartile range, IQR) and evaluated via the non-parametric Mann–Whitney U test. Qualitative parameters were expressed as frequencies and proportions, with intergroup differences evaluated via Pearson’s chi-square test. To identify factors independently associated with PAS, we conducted univariable and multivariable Firth penalized logistic regressions as the primary analyses to reduce small-sample bias and improve the stability of the estimates. Conventional maximum-likelihood logistic regression was conducted as a sensitivity analysis. As exploratory sensitivity analyses, regularized logistic regression models—specifically the least absolute shrinkage and selection operator (LASSO), ridge, and Elastic Net—were additionally implemented to assess the robustness of our findings under coefficient shrinkage. Spearman’s rank correlation analysis was used to assess the relationships between left CAVI, right CAVI, serum OPN levels, and clinical variables. We evaluated the predictive discrimination of the regression models for PAS via receiver operating characteristic analysis and reported the area under the curve (AUC). Ninety-five percent confidence intervals (95% CIs) for the AUCs were derived from 5000 stratified bootstrap resamples, with optimal cutoff points determined according to the maximum Youden index. Predictive calibration was determined through the Brier score, Hosmer–Lemeshow goodness-of-fit statistic, and assessments of calibration intercept and slope. Model performance was internally validated using 5000 stratified bootstrap resamplings to calculate optimism-adjusted AUCs, Brier scores, and calibration metrics (intercept and slope). Statistical significance was defined as a two-tailed *p*-value < 0.05.

## 3. Results

### 3.1. Baseline Clinical Characteristics of the Study Population

The clinical and biochemical characteristics of the 120 patients with T2DM, stratified by the presence of PAS (CAVI ≥ 9.0, *n* = 72) or absence of PAS (CAVI < 9.0, *n* = 48), are presented in Table 1. Patients with PAS were significantly older than those without PAS (*p* < 0.001) and, as expected, had significantly higher left and right CAVI values (*p* < 0.001). Regarding metabolic and renal profiles, patients with PAS exhibited poorer glycemic control, as indicated by significantly higher fasting glucose (*p* = 0.015) and HbA1c (*p* = 0.006) levels. They also had significantly lower HDL-C (*p* = 0.021) and higher UACR (*p* = 0.007) and OPN levels (*p* < 0.001). Conversely, no significant intergroup differences were detected between the two groups regarding sex distribution, BMI, BP, TCH, TG, LDL-C, eGFR, calcium, phosphorus, intact parathyroid hormone, or the use of antihypertensive and antidiabetic medications.

### 3.2. Factors Associated with PAS

Variables that differed significantly between the two groups in Table 1 were subsequently evaluated using univariable Firth penalized logistic regression (Table 2). Higher OPN levels (odds ratio [OR], 1.045 per 1-ng/mL increase; 95% CI, 1.024–1.071; *p* < 0.001), older age (OR, 1.091 per 1-year increase; 95% CI, 1.046–1.145; *p* < 0.001), higher fasting glucose levels (OR, 1.014 per 1-mg/dL increase; 95% CI, 1.005–1.026; *p* = 0.003), and higher HbA1c levels (OR, 1.872 per 1% increase; 95% CI, 1.285–2.954; *p* < 0.001) were significantly associated with PAS. In contrast, higher HDL-C levels were associated with lower odds of PAS (OR, 0.957 per 1-mg/dL increase; 95% CI, 0.916–0.994; *p* = 0.022). All six candidate variables were simultaneously entered in the multivariable Firth penalized logistic regression model. Higher OPN levels (adjusted OR [aOR], 1.035; 95% CI, 1.012–1.066; *p* = 0.001) and older age (aOR, 1.096; 95% CI, 1.043–1.161; *p* < 0.001) remained independently associated with PAS, whereas higher HDL-C levels remained independently associated with lower odds of PAS (aOR, 0.946; 95% CI, 0.895–0.992; *p* = 0.021). Fasting glucose, HbA1c, and UACR were not independently associated with PAS after multivariable adjustment. Sensitivity analysis using conventional multivariable logistic regression produced consistent results, confirming independent associations for OPN (aOR, 1.042; 95% CI, 1.014–1.071; *p* = 0.003), age (aOR, 1.104; 95% CI, 1.045–1.167; *p* < 0.001), and HDL-C (aOR, 0.940; 95% CI, 0.890–0.993; *p* = 0.026), thereby supporting the robustness of the primary findings. Variance inflation factors for all variables in the original multivariable model ranged from 1.10 to 1.87, indicating no evidence of substantial multicollinearity. Additional adjustment for biological sex did not materially alter the inverse association between HDL-C and PAS (Supplementary Table S1).

### 3.3. Sensitivity Analysis Using LASSO, Ridge, and Elastic Net Logistic Regression

Exploratory LASSO, ridge, and Elastic Net logistic regression analyses yielded directionally consistent estimates for OPN, age, and HDL-C (Table 3). The regularized odds ratios for OPN per 1 ng/mL increase were 1.032, 1.028, and 1.030, respectively. Corresponding estimates were 1.086, 1.081, and 1.084 for age and 0.956, 0.954, and 0.955 for HDL-C. These findings indicate that the directions of the estimated associations were maintained under different regularization penalties. These analyses were interpreted descriptively and were not used to establish additional statistically significant associations.

### 3.4. Correlations of CAVI and Serum Osteopontin with Clinical Variables

Left CAVI was positively correlated with age (ρ = Spearman’s rank correlation coefficient; ρ = 0.433, *p* < 0.001), right CAVI (ρ = 0.862, *p* < 0.001), OPN levels (ρ = 0.381, *p* < 0.001), fasting glucose (ρ = 0.220, *p* = 0.016), HbA1c (ρ = 0.314, *p* < 0.001), and UACR (ρ = 0.264, *p* = 0.004). In contrast, left CAVI was inversely correlated with HDL-C levels (ρ = −0.282, *p* = 0.002). Similarly, right CAVI was positively correlated with age (ρ = 0.434, *p* < 0.001), left CAVI (ρ = 0.862, *p* < 0.001), OPN levels (ρ = 0.418, *p* < 0.001), HbA1c (ρ = 0.232, *p* = 0.011), and UACR (ρ = 0.287, *p* = 0.001). Right CAVI was also inversely correlated with HDL-C levels (ρ = −0.188, *p* = 0.040). Serum OPN levels were positively correlated with age (ρ = 0.274, *p* = 0.002), BMI (ρ = 0.188, *p* = 0.040), left CAVI (ρ = 0.381, *p* < 0.001), and right CAVI (ρ = 0.418, *p* < 0.001). In contrast, OPN levels were inversely correlated with total calcium levels (ρ = −0.248, *p* = 0.006) (Table 4).

### 3.5. Discrimination and Calibration of Models for PAS

Among the individual prediction models, OPN demonstrated the highest discriminatory performance for PAS, with an AUC of 0.748 (95% CI, 0.651–0.834; *p* < 0.001), followed by age (AUC, 0.720; 95% CI, 0.620–0.813; *p* < 0.001) and HDL-C (AUC, 0.625; 95% CI, 0.526–0.718; *p* = 0.013). The baseline model containing age and HDL-C yielded an AUC of 0.772 (95% CI, 0.685–0.856; *p* < 0.001), a sensitivity of 0.722, a specificity of 0.729, and a Brier score of 0.187. Following bootstrap correction, this baseline model had an optimism-corrected AUC of 0.765, a Brier score of 0.194, a calibration intercept of 0.005, and a calibration slope of 0.952. The extended model incorporating OPN, age, and HDL-C achieved the highest apparent discriminatory performance, with an AUC of 0.831 (95% CI, 0.756–0.899; *p* < 0.001), a sensitivity of 0.792, a specificity of 0.750, and a Brier score of 0.163. Following bootstrap correction, the model maintained good performance, with an optimism-corrected AUC of 0.817, a Brier score of 0.173, a calibration intercept of 0.005, and a calibration slope of 0.918. Compared with the age and HDL-C baseline model, adding OPN increased the apparent AUC by 0.059 and the optimism-corrected AUC by 0.052. The corresponding apparent and optimism-corrected Brier scores decreased from 0.187 to 0.163 and from 0.194 to 0.173, respectively. The Hosmer–Lemeshow goodness-of-fit test indicated no evidence of poor calibration for the age-only, HDL-C-only, age and HDL-C baseline, or OPN, age, and HDL-C extended models (*p* = 0.834, 0.522, 0.557, and 0.370, respectively). In contrast, the OPN-only model showed evidence of miscalibration (*p* < 0.001, Table 5).

## 4. Discussion

In this cross-sectional study of 120 patients with T2DM, 60.0% met the CAVI-based criterion for PAS. Serum OPN levels were significantly higher in patients with PAS and were positively correlated with both left and right CAVI values. After adjustment for age, HDL-C, fasting glucose, HbA1c, and UACR using Firth penalized logistic regression, higher OPN levels remained independently associated with PAS, along with older age and lower HDL-C levels. The consistency of these associations across conventional, LASSO, ridge, and Elastic Net logistic regression models further supports the robustness of the findings. Although OPN alone demonstrated moderate discrimination for PAS, combining OPN with age and HDL-C improved model discrimination while maintaining acceptable calibration after bootstrap correction. Collectively, these findings suggest that serum OPN may be a useful adjunctive biomarker for identifying patients with T2DM at increased risk of PAS.

The association between older age and PAS likely reflects age-related elastin degradation and collagen cross-linking within the arterial wall [13,14]. This finding is consistent with previous evidence showing that age-related extracellular matrix remodeling and interactions with vascular smooth muscle cells (VSMCs) contribute to vascular senescence and chronic inflammation [15]. Conversely, the inverse association between HDL-C and PAS may be attributable to the pleiotropic vascular effects of HDL-C, including enhanced nitric oxide bioavailability, attenuation of subclinical inflammation, and promotion of reverse cholesterol transport, all of which help preserve endothelial integrity [16,17]. The unadjusted associations of glucose-related markers and UACR with PAS may reflect the complex interplay between metabolic dysregulation and vascular remodeling [18,19]. Chronic hyperglycemia promotes oxidative stress and the formation of advanced glycation end products, leading to endothelial dysfunction and VSMC proliferation [20,21]. In addition, albuminuria is a recognized marker of endothelial dysfunction in patients with T2DM [22]. Accordingly, elevated UACR may indicate systemic endothelial dysfunction, with renal microvascular injury paralleling the macrovascular stiffness measured by CAVI, thereby reflecting the shared vascular vulnerability associated with T2DM [22,23,24].

The bone–vascular axis concept recognizes that regulators of bone metabolism, including OPN, play pivotal roles in vascular pathophysiology [25,26]. OPN is a phosphorylated glycoprotein initially identified in bone tissue that regulates mineralization in both skeletal and vascular systems [25,26]. The mechanisms underlying these relationships are complex and, within the context of human observational studies, largely inferred from experimental paradigms. OPN is markedly upregulated in response to vascular injury and chronic inflammation, functioning as a matricellular protein involved in tissue remodeling [27]. Experimental studies have shown that OPN can facilitate the phenotypic transition of VSMCs toward an osteoblast-like phenotype, thereby potentially contributing to medial arterial calcification [28,29,30]. Although OPN inhibits hydroxyapatite crystal formation, elevated circulating OPN levels in patients with higher CAVI values are hypothesized to represent a compensatory response to ongoing vascular calcification and extracellular matrix remodeling [29,30].

The positive correlation between OPN and age may be explained by the process of inflammaging [31]. During aging, senescent vascular cells develop a senescence-associated secretory phenotype (SASP), characterized by increased secretion of proinflammatory cytokines and OPN [32,33,34,35]. Beyond serving as a component of the SASP, OPN has been implicated in experimental models of the phenotypic transition of VSMCs from a contractile to a synthetic, senescent phenotype [36]. Elevated OPN levels have also been reported to correlate with proinflammatory signaling and downstream matrix metalloproteinase activation, which may promote elastin degradation and extracellular matrix stiffening in laboratory settings [37]. Collectively, these age-related molecular alterations contribute to systemic vascular remodeling and may underlie the development of macrovascular stiffness in aging and metabolic diseases.

The weak inverse correlation between OPN and total calcium observed in this study may reflect the complex interplay between OPN and mineral homeostasis [27]. Under conditions of elevated calcium concentrations or local mineral imbalance, OPN is recruited to the arterial wall, where it binds hydroxyapatite crystals and acts as a potent inhibitor of crystal nucleation by sequestering calcium–phosphate minerals, thereby limiting ectopic calcification [38]. Through its highly acidic, phosphorylated domains, OPN suppresses the growth of mineral deposits [39]. This phenomenon is consistent with the calcification paradox, in which pathways involved in bone remodeling also contribute to vascular mineralization [6,27,40]. In addition, OPN may promote macrophage infiltration through its arginine–glycine–aspartic acid motif, potentially exacerbating vascular remodeling despite its protective inhibitory role within the proinflammatory milieu of T2DM [7]. Together, these findings suggest that circulating OPN reflects multiple vascular and metabolic processes associated with CAVI-defined arterial stiffness in patients with T2DM [6,7,27,31,38,40].

The strengths of this study include standardized bilateral CAVI measurements, the use of Firth penalized logistic regression complemented by multiple sensitivity analyses, and a comprehensive evaluation of model discrimination and calibration with internal bootstrap validation. Nevertheless, several limitations should be acknowledged. First, the cross-sectional design precludes inference of temporal or causal relationships. Consequently, whether elevated circulating OPN directly contributes to arterial wall stiffening or represents a compensatory response to ongoing mechanical and mineral stress remains unresolved. The observed associations between OPN, HDL-C, and CAVI-defined PAS should therefore be interpreted strictly as cross-sectional markers of concurrent vascular vulnerability rather than evidence that these biomarkers influence disease progression or predict prospective macrovascular events. Second, this single-center study included a relatively small sample of 120 patients with T2DM and lacked a nondiabetic comparison group. This absence precludes direct comparative analyses to assess whether the relationship between OPN and arterial stiffness differs across glycemic states, limits our ability to establish normative baseline reference thresholds for OPN, and restricts the generalizability of our findings to broader or non-diabetic populations. Furthermore, while candidate covariates were selected based on both biological plausibility across diabetic vascular axes and between-group differences, certain clinically important determinants could not be incorporated. Although patients with overt cardiovascular history (such as acute coronary syndrome or heart failure) were preemptively excluded and baseline treatment exposures (antihypertensive, lipid-lowering, and hypoglycemic agents) were well-balanced between groups, granular clinical and lifestyle factors—most notably exact diabetes duration from initial diagnosis, cigarette smoking status, quantitative physical activity levels, and medication adherence—were not systematically documented in the current cohort. Given that prolonged glycemic exposure, tobacco use, physical inactivity, and suboptimal therapy adherence are established contributors to vascular senescence and arterial stiffening, residual confounding from these unmeasured clinical and lifestyle factors cannot be fully excluded. Additionally, while the CAVI ≥ 9.0 cutoff is derived from Japanese guidelines and widely applied across East Asian cohorts, whether a population-specific threshold tailored specifically to Taiwanese patients with T2DM would alter risk classification remains to be prospectively validated. Nonetheless, the consistent positive correlations between serum OPN and continuous bilateral CAVI values indicate that our primary findings are not an artifact of binary threshold categorization. Third, OPN was measured only once. Because OPN is a nonspecific systemic biomarker influenced by inflammation and bone metabolism, a single measurement cannot establish vascular specificity or account for intraindividual variability. Furthermore, our study did not directly assess vascular calcification (e.g., via computed tomography calcium scoring), phenotypic VSMC transdifferentiation markers, or circulating MMP enzymatic activities; consequently, the proposed cellular and molecular mechanisms connecting OPN with arterial wall stiffening discussed herein are extrapolated from experimental literature and remain hypothetical. Fourth, routinely used clinical inflammatory markers, such as C-reactive protein (CRP), were not measured in our study cohort. Although OPN captures active local arterial remodeling and vascular calcification pathways distinct from hepatic acute-phase responses, the lack of CRP precluded direct comparisons regarding the incremental discriminative value of OPN over standard systemic inflammatory markers. Fifth, the Spearman correlation analyses included multiple pairwise comparisons without adjustment for multiple testing and should therefore be interpreted as exploratory. Finally, although bootstrap correction demonstrated acceptable discrimination and calibration for the combined OPN, age, and HDL-C model, this represented internal validation only because model development and evaluation were conducted within the same cohort. Moreover, the OPN-only model exhibited poor calibration, indicating that OPN should not be used as a standalone clinical classifier. Accordingly, the present findings demonstrate cross-sectional discriminatory ability rather than established clinical utility or prospective predictive value. Larger, multicenter longitudinal studies incorporating prespecified covariates, repeated OPN measurements, and external model validation are needed to determine whether OPN improves risk stratification for CAVI-defined PAS and future vascular outcomes.

## 5. Conclusions

In patients with T2DM, higher serum OPN levels were independently associated with the presence of CAVI-defined PAS and were positively correlated with bilateral CAVI values. Combining OPN with age and HDL-C improved discriminatory performance compared with OPN alone while maintaining acceptable performance after internal bootstrap validation. These findings suggest that OPN may serve as an adjunctive cross-sectional biomarker associated with prevalent PAS but should not be used as a standalone clinical classifier. Because causality and directionality cannot be inferred from the current cross-sectional design, larger prospective, multicenter longitudinal studies are warranted to determine its incremental clinical value and ability to predict future vascular outcomes.

## Figures and Tables

**Table 1:** Clinical characteristics of patients with type 2 diabetes mellitus stratified by peripheral arterial stiffness (CAVI ≥ 9.0) or the control group (CAVI < 9.0).

Characteristic	All Participants (*n* = 120)	Control Group (*n* = 48)	PAS Group (*n* = 72)	*p* Value
Age (years)	64.03 ± 10.54	59.00 ± 10.94	67.39 ± 8.85	<0.001 *
Body mass index (kg/m^2^)	28.59 ± 3.91	28.01 ± 3.81	28.97 ± 3.96	0.191
Left CAVI	9.21 ± 1.55	7.80 ± 1.23	10.15 ± 0.97	<0.001 *
Right CAVI	9.06 ± 1.41	7.80 ± 1.03	9.90 ± 0.91	<0.001 *
SBP (mmHg)	147.66 ± 22.09	148.25 ± 27.22	147.26 ± 18.08	0.812
DBP (mmHg)	86.08 ± 10.29	87.40 ± 11.63	85.19 ± 9.27	0.253
Total cholesterol (mg/dL)	157.53 ± 26.20	156.65 ± 26.46	158.13 ± 26.19	0.763
Triglyceride (mg/dL)	138.50 (96.25–176.25)	138.50 (91.25–175.50)	139.00 (101.25–180.75)	0.714
HDL-C (mg/dL)	47.83 ± 9.90	50.38 ± 8.27	46.13 ± 10.57	0.021 *
LDL-C (mg/dL)	91.85 ± 25.21	91.50 ± 23.67	92.08 ± 26.35	0.902
Albumin (g/dL)	4.30 (4.20–4.50)	4.35 (4.13–4.60)	4.30 (4.20–4.50)	0.278
Fasting glucose (mg/dL)	140.00 (121.00–169.75)	134.50 (118.25–152.75)	150.00 (122.75–185.25)	0.015 *
Glycated hemoglobin (%)	7.50 (7.00–8.40)	7.50 (6.90–7.80)	7.85 (7.10–8.80)	0.006 *
Blood urea nitrogen (mg/dL)	18.00 (14.00–22.00)	18.00 (14.25–24.00)	17.00 (14.00–22.00)	0.399
Creatinine (mg/dL)	1.00 (0.80–1.30)	0.95 (0.80–1.30)	1.00 (0.80–1.30)	0.419
eGFR (mL/min)	73.20 ± 21.41	75.59 ± 23.73	71.61 ± 19.80	0.321
Total calcium (mg/dL)	9.09 ± 0.39	9.13 ± 0.39	9.07 ± 0.40	0.437
Phosphorus (mg/dL)	3.56 ± 0.54	3.58 ± 0.59	3.55 ± 0.50	0.766
UACR (mg/g)	45.03 (32.76–138.15)	35.39 (26.69–93.67)	54.82 (36.04–177.70)	0.007 *
Osteopontin (ng/mL)	35.45 (22.57–51.93)	26.07 (9.12–44.89)	38.37 (31.81–67.05)	<0.001 *
iPTH (pg/mL)	12.77 (8.80–21.64)	12.45 (8.84–20.34)	12.77 (8.77–22.56)	0.991
Male, *n* (%)	78 (65.0)	28 (58.3)	50 (69.4)	0.211
Hypertension, *n* (%)	81 (67.5)	32 (66.7)	49 (68.1)	0.874
ACEi/ARB use, *n* (%)	75 (62.5)	28 (58.3)	47 (65.3)	0.441
β-blocker use, *n* (%)	24 (20.0)	9 (18.8)	15 (20.8)	0.780
CCB use, *n* (%)	44 (36.7)	20 (41.7)	24 (33.3)	0.353
Statin use, *n* (%)	60 (50.0)	23 (47.9)	37 (51.4)	0.709
Fibrate use, *n* (%)	17 (14.2)	5 (10.4)	12 (16.7)	0.336
Metformin use, *n* (%)	72 (60.0)	27 (56.3)	45 (62.5)	0.494
Sulfonylurea use, *n* (%)	65 (54.2)	25 (52.1)	40 (55.6)	0.708
DPP-4 inhibitor use, *n* (%)	70 (58.3)	25 (52.1)	45 (62.5)	0.257
Insulin use, *n* (%)	46 (38.3)	21 (43.8)	25 (34.7)	0.319
SGLT2i, *n* (%)	33 (27.5)	15 (31.3)	18 (25.0)	0.453

Continuous variables are presented as the mean ± standard deviation and were compared using Student’s *t*-test; skewed continuous data are reported as medians with interquartile ranges and analyzed via the non-parametric Mann–Whitney U test. Qualitative parameters are summarized as counts and proportions (*n*, %), with intergroup differences evaluated via Pearson’s chi-square test. ACEi, angiotensin-converting enzyme inhibitor; ARB, angiotensin receptor blocker; CAVI, cardio-ankle vascular index; CCB, calcium channel blocker; DBP, diastolic blood pressure; DPP-4i, dipeptidyl peptidase-4 inhibitor; eGFR, estimated glomerular filtration rate; HDL-C, high-density lipoprotein cholesterol; iPTH, intact parathyroid hormone; LDL-C, low-density lipoprotein cholesterol; PAS, peripheral arterial stiffness; SBP, systolic blood pressure; SGLT2i, sodium–glucose cotransporter 2 inhibitor; UACR, urine albumin-to-creatinine ratio. * *p* < 0.05 indicates statistical significance.

**Table 2:** Determinants of peripheral arterial stiffness in patients with type 2 diabetes mellitus: Primary Firth penalized logistic regression and conventional logistic sensitivity analyses.

Variables	Univariable Firth Penalized Logistic Regression		Multivariable Firth Penalized Logistic Regression		Conventional Multivariable Logistic Regression	
	OR (95% CI)	*p* Value	aOR (95% CI)	*p* Value	aOR (95% CI)	*p* Value
Osteopontin, per 1 ng/mL increase	1.045 (1.024–1.071)	<0.001 *	1.035 (1.012–1.066)	0.001 *	1.042 (1.014–1.071)	0.003 *
Age, per 1-year increase	1.091 (1.046–1.145)	<0.001 *	1.096 (1.043–1.161)	<0.001 *	1.104 (1.045–1.167)	<0.001 *
HDL-C, per 1 mg/dL increase	0.957 (0.916–0.994)	0.022 *	0.946 (0.895–0.992)	0.021 *	0.940 (0.890–0.993)	0.026 *
Fasting glucose, per 1 mg/dL increase	1.014 (1.005–1.026)	0.003 *	1.003 (0.987–1.020)	0.725	1.004 (0.987–1.022)	0.627
HbA1c, per 1% increase	1.872 (1.285–2.954)	<0.001 *	1.722 (0.964–3.364)	0.067	1.843 (0.967–3.511)	0.063
UACR: per 1 g/g increase	1.578 (0.749–7.678)	0.282	0.913 (0.376–9.524)	0.906	1.718 (0.259–11.390)	0.575

The primary analyses were performed using univariable and multivariable Firth penalized logistic regression. The multivariable model simultaneously included age, fasting glucose, HbA1c, HDL-C, UACR, and osteopontin. Conventional maximum-likelihood multivariable logistic regression using the same covariates was performed only as a sensitivity analysis. Firth-model 95% confidence intervals and *p*-values were based on penalized profile likelihood; conventional-model confidence intervals and *p*-values were Wald-based. Abbreviations: OR, odds ratio; aOR, adjusted odds ratio; CI, confidence interval; HbA1c, glycated hemoglobin; HDL-C, high-density lipoprotein cholesterol; UACR, urine albumin-to-creatinine ratio. * *p* < 0.05 was considered statistically significant.

**Table 3:** Exploratory sensitivity analyses using LASSO, ridge, and Elastic Net logistic regression for peripheral arterial stiffness in patients with type 2 diabetes mellitus.

Factors	LASSO	Ridge	Elastic Net
	Regularized OR	Regularized OR	Regularized OR
Osteopontin, per 1 ng/mL increase	1.032	1.028	1.030
Age, per 1-year increase	1.086	1.081	1.084
HDL-C, per 1 mg/dL increase	0.956	0.954	0.955
Fasting glucose, per 1 mg/dL increase	1.002	1.005	1.004
HbA1c, per 1% increase	1.627	1.524	1.576
UACR: per 1 g/g increase	1.000	1.408	1.151

All models included age, HDL-C, fasting glucose, HbA1c, UACR, and osteopontin as candidate predictors. Predictors were standardized before model fitting, and odds ratios are reported in their original measurement units. Penalty strength was selected using stratified 10-fold cross-validation to minimize binomial deviance. The Elastic Net mixing parameter was fixed at α = 0.5. These models were used as exploratory sensitivity analyses to assess the direction and magnitude of associations under coefficient shrinkage. Only regularized odds-ratio estimates are reported; confidence intervals and *p*-values are not presented because a validated coefficient-level inferential procedure was not established for these analyses. Primary inferential results from Firth penalized logistic regression are reported in Table 2. Abbreviations: CI, confidence interval; HDL-C, high-density lipoprotein cholesterol; HbA1c, glycated hemoglobin; UACR, urine albumin-to-creatinine ratio; OR, odds ratio.

**Table 4:** Spearman correlation coefficients between left cardio-ankle vascular index, right cardio-ankle vascular index, osteopontin, and clinical variables in patients with type 2 diabetes mellitus.

Variables	Left CAVI		Right CAVI		Osteopontin (ng/mL)	
	Spearman Correlation Coefficient	*p* Value	Spearman Correlation Coefficient	*p* Value	Spearman Correlation Coefficient	*p* Value
Age (years)	0.433	<0.001 *	0.434	<0.001 *	0.274	0.002 *
Body mass index (kg/m^2^)	0.115	0.212	0.103	0.263	0.188	0.040 *
Left CAVI	—	—	0.862	<0.001 *	0.381	<0.001 *
Right CAVI	0.862	<0.001 *	—	—	0.418	<0.001 *
Osteopontin (ng/mL)	0.381	<0.001 *	0.418	<0.001 *	—	—
SBP (mmHg)	0.133	0.147	0.070	0.450	0.025	0.784
DBP (mmHg)	−0.043	0.645	−0.132	0.150	−0.034	0.712
TCH (mg/dL)	−0.057	0.539	−0.030	0.742	−0.142	0.123
Triglyceride (mg/dL)	0.122	0.183	0.054	0.555	0.168	0.067
HDL-C (mg/dL)	−0.282	0.002 *	−0.188	0.040 *	−0.043	0.641
LDL-C (mg/dL)	−0.098	0.287	−0.037	0.692	0.059	0.524
Glucose (mg/dL)	0.220	0.016 *	0.164	0.073	0.036	0.694
HbA1c (%)	0.314	<0.001 *	0.232	0.011 *	0.119	0.195
BUN (mg/dL)	−0.115	0.211	−0.104	0.259	−0.014	0.876
Creatinine (mg/dL)	0.110	0.232	0.115	0.212	0.073	0.431
eGFR (mL/min)	−0.069	0.456	−0.068	0.457	−0.008	0.930
UACR (mg/g)	0.264	0.004 *	0.287	0.001 *	0.163	0.075
Total calcium (mg/dL)	−0.059	0.525	−0.091	0.325	−0.248	0.006 *
Phosphorus (mg/dL)	0.013	0.890	−0.019	0.838	−0.114	0.215
iPTH (pg/mL)	0.017	0.852	0.076	0.408	0.168	0.067

Correlations among continuous variables were evaluated using Spearman's rank correlation analysis. Abbreviations: BUN, blood urea nitrogen; CAVI, cardio-ankle vascular index; DBP, diastolic blood pressure; eGFR, estimated glomerular filtration rate; HbA1c, glycated hemoglobin; HDL-C, high-density lipoprotein cholesterol; iPTH, intact parathyroid hormone; LDL-C, low-density lipoprotein cholesterol; SBP, systolic blood pressure; TCH, total cholesterol; UACR, urine albumin-to-creatinine ratio. “—" indicates not applicable, as the correlation of a variable with itself was not calculated. * *p* < 0.05 was considered statistically significant.

**Table 5:** Discrimination and calibration of models in 120 type 2 diabetic patients with peripheral arterial stiffness.

Outcome	Model	Events /Total	AUC (95% CI) *p* Value	Sensitivity	Specificity	Brier Score	Hosmer- Lemeshow *p*-Value	Bootstrap- Corrected AUC	Bootstrap- Corrected Brier Score	Bootstrap-Corrected Calibration Intercept	Bootstrap Corrected the Calibration Slope
Peripheral arterial stiffness	Osteopontin	72/120	0.748 (0.651–0.834) <0.001 *	0.847	0.583	0.196	<0.001 *	0.748	0.199	−0.006	1.017
	Age		0.720 (0.620–0.813) <0.001 *	0.819	0.604	0.202	0.834	0.720	0.205	−0.002	1.039
	HDL-C		0.625 (0.526–0.718) 0.013 *	0.458	0.813	0.230	0.522	0.623	0.234	0.008	1.102
	Age + HDL-C		0.772 (0.685–0.856) <0.001 *	0.722	0.729	0.187	0.557	0.765	0.194	0.005	0.952
	Osteopontin + age + HDL-C		0.831 (0.756–0.899) <0.001 *	0.792	0.750	0.163	0.370	0.817	0.173	0.005	0.918

AUC values were derived from the predicted probabilities generated by the logistic regression models. Ninety-five percent confidence intervals (95% CIs) for the apparent AUCs were estimated using 5000 stratified bootstrap resamples. The AUC *p*-values test the null hypothesis that AUC = 0.50. Optimal cutoffs were selected using the Youden index; cutoffs for the individual models are shown in the original measurement units, whereas the combined-model cutoff is a predicted probability. The Hosmer–Lemeshow test used 10 risk groups. Internal validation via 5000 stratified bootstrap iterations yielded optimism-adjusted AUC, Brier score, calibration intercept, and calibration slope, with models refit in each bootstrap draw and tested in the original dataset. Abbreviations: AUC, area under the receiver operating characteristic curve; CI, confidence interval; HDL-C, high-density lipoprotein cholesterol. * *p* < 0.05 indicated statistical significance.

## Data Availability

The data presented in this study are available upon request from the corresponding authors. The data are not publicly available due to privacy and ethical restrictions involving patient confidentiality.

## Supplementary Materials

The following supporting information can be downloaded at https://www.mdpi.com/article/10.3390/diagnostics16203300/s1. Table S1: Additional sex-adjusted analyses of peripheral arterial stiffness and continuous cardio-ankle vascular index in patients with type 2 diabetes mellitus.

## Abbreviations

The following abbreviations are used in this manuscript:

aOR	Adjusted odds ratio
AUC	Area under the curve
BMI	Body mass index
BP	Blood pressure
CAVI	Cardio-ankle vascular index
CI	Confidence interval
CKD-EPI	Chronic Kidney Disease Epidemiology Collaboration
CRP	C-reactive protein
CV	Cardiovascular
CVD	Cardiovascular disease
DBP	Diastolic blood pressure
eGFR	Estimated glomerular filtration rate
HbA1c	Glycated hemoglobin
HDL-C	High-density lipoprotein cholesterol
LDL-C	Low-density lipoprotein cholesterol
LASSO	Least absolute shrinkage and selection operator
OPN	Osteopontin
OR	Odds ratio
PAS	Peripheral arterial stiffness
PWV	Pulse wave velocity
SASP	Senescence-associated secretory phenotype
SBP	Systolic blood pressure
TCH	Total cholesterol
T2DM	Type 2 diabetes mellitus
TG	Triglyceride
UACR	Urine albumin-to-creatinine ratio
VSMCs	Vascular smooth muscle cells
