# Supplementary material for: Serum Osteopontin Is Associated with Cardio-Ankle Vascular Index-Defined Arterial Stiffness in Patients with Type 2 Diabetes Mellitus: A Cross-Sectional Study
Source: Diagnostics (Basel). 2026 Oct 11;16(20):3300. doi: 10.3390/diagnostics16203300 (PMC13703529; doi:10.3390/diagnostics16203300)
Supplement: Supplementary file 1 [file diagnostics-16-03300-s001.zip › diagnostics-4550282-supplementary.pdf]

Supplementary Table S1. Additional sex-adjusted analyses of peripheral arterial stiffness and continuous cardio-ankle vascular index in patients with type 2 diabetes mellitus.

Variable	Multivariable Firth penalized logistic regression		Conventional multivariable logistic regression	
	aOR (95% CI)	*p* Value	aOR (95% CI)	*p* Value
Osteopontin, per 1 ng/mL	1.034 (1.011–1.065)	0.002*	1.041 (1.012–1.070)	0.005*
Age, per 1 year	1.097 (1.043–1.163)	<0.001*	1.106 (1.045–1.171)	<0.001*
HDL-C, per 1 mg/dL	0.948 (0.896–0.993)	0.023*	0.941 (0.891–0.993)	0.028*
Fasting glucose, per 1 mg/dL	1.003 (0.987–1.020)	0.725	1.004 (0.987–1.022)	0.627
HbA1c, per 1 percentage point	1.691 (0.944–3.306)	0.079	1.818 (0.949–3.481)	0.071
UACR, per 1 g/g	0.921 (0.379–9.874)	0.914	1.760 (0.257–12.046)	0.564
Male sex vs. female	1.183 (0.442–3.135)	0.735	1.216 (0.435–3.396)	0.709

Firth-model 95% CIs were obtained using profile penalized likelihood, and *p*-values using penalized likelihood-ratio tests. Conventional-model CIs and p-values were Wald-based. UACR was expressed per 1 g/g. Continuous predictors were scaled internally for numerical stability; reported aORs were transformed back to the stated units. Abbreviations: aOR, adjusted odds ratio; CAVI, cardio-ankle vascular index; CI, confidence interval; HbA1c, glycated hemoglobin; HDL-C, high-density lipoprotein cholesterol; PAS, peripheral arterial stiffness; UACR, urine albumin-to-creatinine ratio. *$p < 0.05$ was considered statistically significant.
